# Therapeutic Application of Nitrous Oxide

**Published:** 1891-03

**Authors:** 


### Therapeutic Application of Nitrous Oxide.

Neuralgia, uncomplicated, will sometimes be relieved by a few inhalations of nitrous oxide gas.

Nervous Aphonia. This peculiar form of loss of the power over the voice, usually the result of hysteria, will be much improved by the patient inhaling a sufficient amount of the nitrous oxide gas to produce a partial loss of sensation and muscular relaxation.

Local Paralysis has been benefitted, when there was no brain lesion, by the gentle stimulation by the first stages of the gas or the tingling and stimulating effect on the muscles.

Asthma.—This disease, when of a spasmodic character, is often much improved by causing the patient to pass into the stage of relaxation, employing it every other day for a week or two.

Epilepsy.—When this disease is not the result of an organic change in the brain, spine, or other portion of the nervous system, but the result of some peripheral, or reflex action, benefit will ensue by the use of the gas for weeks. It should be administered two or three times a week only, to produce the stimulating effects of the first stage of anæsthesia.

My friend, Dr. George J. Zigler, has found the solution of the gas in water of much utility in diseases of the lungs, kidneys, and other diseases of this class.

In connection with the above we add a part of a discussion of a paper by Dr. John Aulde, M.D., Philadelphia, on Instruments and Appliances for Administering Oxygen. In the discussion of the paper Dr. M. Price said :

" I would like to ask to what the benefit from the use of nitrous oxide is attributed ? Fifteen years ago we used nitrous oxide at the dispensary for the extraction of teeth. Many consumptive cases in which it was used came back in a few weeks stating that they had never been so much benefitted as from the inhalation of nitrous oxide. My explanation is that the nitrous oxide, acting as an anæsthetic, lessens the pain of respiration, and the patient in his efforts to secure air expands the lungs filling portions of them that have not been used. The action is just the same as when we break up the adhesions about an ankylosed joint under ether. I have employed the nitrous oxide in many cases of phthisis with advantage, and dentists have informed me that they have often been told by consumptive patients that they have been benefitted by the inhalation of nitrous oxide."—*Turnbull's Anaesthetic Manual.*

THE OVERWORKED PHYSICIAN'S LUNCHEON.—Dr. Allen McLean Hamilton contributes to the *Dietetic Gazette* some dietetic suggestions in nervous and mental diseases, one of which will interest all those busy practitioners who give themselves no time for a midday repast. His advice would be to lay in a goodly supply of fresh almonds, and to have some of them constantly within reach and to eat freely of them during the spare moments. He writes as follows:

Acting upon a hint given by my friend, Dr. Lauder Brunton, I have directed some of my patients to eat freely of fresh almonds, which are rich in oil and exceedingly nutritious, containing as they do 54 per cent. of fixed oil. According to Pavy they contain 2.677 of nitrogen and 40 per cent. of carbon. It is a custom of Dr. Brunton and several other London physicians, when hurried and tired after their morning consulting hours, to make a luncheon simply of this kind. In cases of diabetes, when digestion is not too weak, it will be found that biscuits of almond flour are exceedingly nutritious and palatable and may take the place of gluten bread.

MEDICAL PRACTICE IN MINNESOTA.—The results of seven years operation of the Medical Practice Acts in that State have been to reduce the proportion of physicians to 1 in 1,250 persons; whereas it formerly was 1 in 650. Some hundreds of pretenders have been forced to quit the State, and have gone into Michigan and other unprotected States. The present examination act has been in force three years, and in that time only 205 candidates have presented themselves for examination, of these 77, or 36 per cent., were rejected.

